# An on-farm investigation of beef suckler herds using an animal welfare index (AWI)

**DOI:** 10.1186/1746-6148-6-55

**Published:** 2010-12-13

**Authors:** Mickael Mazurek, Daniel J Prendiville, Mark A Crowe, Isabelle Veissier, Bernadette Earley

**Affiliations:** 1 Animal and Bioscience Research Department, Animal & Grassland Research and Innovation Centre, Teagasc, Grange, Dunsany, Co. Meath, Ireland; 2 Animal & Grassland Research and Innovation Centre, Teagasc, Grange, Dunsany, Co. Meath, Ireland; 3 School of Agriculture, Food Science & Veterinary Medicine and the Conway Institute, University College Dublin, Belfield, Dublin 4, Ireland; 4 URH-ACS, I.N.R.A., site de Theix, F-63122, St. Genès Champanelle, France

## Abstract

**Background:**

Beef suckler farms (194 farms throughout 13 counties) were assessed once with housed cattle and once with cattle at grass using an animal welfare index (AWI). Twenty-three of the 194 farms were revisited a year later and re-evaluated using the AWI and the Tier-Gerechtheits-Index 35L/2000 (TGI35L/2000). Thirty-three indicators were collected in five categories: locomotion (5 indicators); social interactions (between animals) (7), flooring (5), environment (7) and Stockpersonship (9). Three indicators relating to the size of the farm were also collected.

Improving animal welfare is an increasingly important aspect of livestock production systems predominantly due to increased consumer concern about the source of animal products. The objectives were (i) to evaluate animal welfare of Irish beef suckler herds using an animal welfare index (AWI), (ii) to examine correlations between parameters, how they influence the AWI and investigate the applicability of the parameters used, (iii) to investigate the impact of the activity of the farmer (full-time or part-time), the interest of the farmer and the number of animals on the AWI.

**Results:**

The mean AWI was 65% and ranged from 54% to 83%. The grazing period represented 16.5% of the total points of the AWI. Seventy percent of the farms were rated as "Very Good" or "Excellent". There was no difference (P > 0.05) in AWI between full-time and part-time farmers. Part-time farmers had greater (P = 0.01) "social interactions": calving (P = 0.03) and weaning (P < 0.001) scores. Full-time farmers had cleaner animals (P = 0.03) and their animals had less lameness (P = 0.01). The number of animals on-farm and the interest of the Stockperson were negatively and positively correlated (P = 0.001), respectively, with the AWI. A hierarchical classification was performed to examine how the indicators influenced the AWI.

**Conclusion:**

The AWI was easily applicable for an on-farm evaluation of welfare. The Stockpersonship was an important factor in determining the AWI (11% of the total variation) more specifically, the interest of the farmer. Part and full-time farming did not differ (P > 0.05) in AWI scores. This method could, with further development, be used in countries with both intensive and/or extensive production systems and would require substantially less resources than animal-based methods.

## Background

Indicators for the assessment of farm animal housing were proposed by several research teams and minimal requirements for animal welfare were implemented in the legislation of most European Union member states (EU directives) as reviewed by von Borell [1]. However, codes of practices for the welfare of farm animals are available (EU code of recommendation; Australian Animal Welfare Standards and Guidelines). In order to assess animal welfare on farms in various production systems, different assessment methods have been developed in Europe [2].

These methods have taken into consideration the advantages and disadvantages of specific housing and management features for the welfare of farm animals. The idea of creating an index system for welfare assessment originates from a concept of Bartussek [3], proposing a Tier-Gerechtheits-Index (TGI, translated as animal needs index) in the context of a state directive for intensive animal housing legislation in Austria. The concept has been further developed leading to the TGI35L/2000 [4,5]. The TGI35L/2000 is a method that assesses the impact of the housing system on animal welfare of cattle, pigs and poultry mainly for organic production. Selected aspects of the animals' environment and farm management are assessed and scored; the higher the score the better the welfare. The scores are summarized to give an overall welfare score. Later, the TGI200 was developed which is similar to the TGI35L/2000 and extends beyond certification and provides advice to farmers [6,7]. More animal-based indicators are included in the TGI200 and the maximum score possible is 200. Other methods utilized mainly animal-based indicators, these methods are more accurate but not practical for on-farm assessment due to the length of time required to complete the inspection [8]. Recently, a new method taking into account animal-based parameters and with a scoring based on a multivariate analysis was developed [9]. The EU funded Welfare Quality^® ^project aimed to accommodate societal concerns and market demands, to develop reliable on-farm monitoring systems, product information systems, and practical species-specific strategies to improve animal welfare. The aims of the Welfare Quality^® ^Project were focused on three main species and their products: cattle (beef and dairy), pigs, and poultry (broiler chickens and laying hens)[1]. After discussions with consumers and scientists, stakeholders, and policy makers, Welfare Quality^® ^defined four animal welfare principles: good housing, good feeding, good health, and appropriate behaviour [2].

During the last few decades European agriculture has changed significantly with increased mechanization and an increase in the number of part-time farmers, so that the time spent by the Stockperson in contact with animals is reduced [10]. These factors and the number of animals that are managed by a Stockperson have been reported to influence human-animal interactions creating welfare (animals) and safety issues (animals and humans) [11]. The welfare, health and management of farm animals are important concerns that need to be addressed in order to increase consumer acceptance of animal production systems in the future. In Ireland, 60% of farmers are presently operating on a part-time basis [12]. There is currently no scientific data available on the status of animal welfare on beef suckler farms in Ireland. The TGI35L/2000 [5] was modified and used to assess animal welfare at farm level.

The objectives of the present study were: (i) to evaluate animal welfare of Irish beef suckler herds using an AWI derived from the TGI35L/2000 [5], (ii) to examine correlations between parameters, how they influence the AWI and investigate the applicability of the parameters used, (iii) to investigate the impact of the activity of the farmer (full-time or part-time), the interest of the farmer and the number of animals on the AWI.

## Methods

### Farm selection

The number of visited farms per county district ranged from 13 to 20. The Agricultural Officer (Head of Advisory Centre) of The National agriculture research and extension organization (Teagasc) for each county in Ireland was contacted to identify suckler beef farms for the AWI assessment. The selected farmers were then contacted by the local Teagasc Adviser to arrange access to the farm for the welfare assessment. A total of 194 farms were visited and data for each indicator were collected.

### Preliminary assessment

Five farms (not included in the study) were selected in a preliminary pilot study to test the repeatability of the assessment and to familiarize the two assessors with scoring of the indicators for use in the main study.

### Farm inspections

One hundred and ninety-four farms were visited to assess the AWI. Farms were visited from March 2006 to April 2007; once during the winter housing period and again at grass during spring. Three indicators of farm size were collected:

i) number of hectares, ii) number of cows, iii) number of animals in the herd (cows, calves, heifers, bulls). In addition, the working status of the farmer (part-time or full-time; full-time implied that they required more than 0.75 labour units to operate; part-time implied that they required less than 0.75 labour units to operate). Two distracter objectives were given to the farmers to ensure they were naïve to the on-farm assessments; 1) to evaluate meal and silage quality and 2) to collect information on the efficacy of vaccines, antibiotics and anthelminthics. The level of interest of the farmer was assessed by a means of a questionnaire.

In March 2009, a second visit of 23 farms out of the 194 was made and data was collected using both the AWI and the TGI35L/2000. The selected farms were located in two counties and animals were housed at the time of the visit. The farms were firstly assessed with the AWI, then with the TGI35L/2000, by two assessors for each method.

### Animal diets during housing

Representative silage and concentrate feed samples were collected from the individual farms during the winter period. In vitro DM digestibility of silage and concentrate feed samples were determined using the method of Tilley and Terry [13].

### AWI indicators

The majority of the indicators listed in the TGI35L/2000 were unchanged, while scores for some indicators were adapted or modified to suit Irish conditions. New indicators were added and irrelevant indicators from the TGI35L/2000 were not used. The AWI grouped 33 indicators into five categories: "locomotion"; five indicators (Table 1), "social interactions"; seven indicators (Table 2), "flooring"; five indicators (Table 3), "environment"; seven indicators (Table 4) and "Stockpersonship"; nine indicators (Table 5). The higher the scores, the better were the conditions regarding animal welfare. The minimum attainable score on the AWI was -11.5; the maximum attainable score was 46, giving a range of 57.5 points. Using the overall score allowed compensation for poor conditions in one category, by better scores in another one, for example a lower score in the "locomotion" category could be compensated for by a better score in the "environment" category. However, a check of the minimal requirements was performed before scoring a farm. Minimum requirements were checked by the two assessors and included the feeding (animals need to be fed every day and in sufficient quantity), drinking (animals need constant water supply) and minimum space allowance.

**Table 1:** Indicators in the AWI of the "Locomotion" category, the definitions used for rating and their maximum individual score.

Score	Space allowance			b)Outdoor access	c) Injurious protrusions	d) Ease of locomotion	e) Grazing time (days per year)
	Slats	Loose housing	Tether systems				
	**(m^2^/AWU)^1^**	**(m^2^/AWU)**	**Movement of tether (m)**				
3.0		> 7.5					> 270
2.5		> 6.5					> 230
2.0		> 5.5		Yes, all the time			> 180
1.5		> 4.5					> 120
1.0	> 3	> 4	> 0.6/0.4	Yes, partially		Easy locomotion	> 50
0.5	2< X^2 ^< 3		> 0.4/0.3				
0	< 2	< 4	< 0.4/0.3	No	No	Partially restraining	
-0.5					Yes	Restraining	

^1^Animal Weight Unit (AWU); 1 AWU = 500 kg live weight. X^2 ^Represents the observed value (X) between 2 and 3 m^2^/AWU. In tether systems, the first figure refers to back and forth movements, the second to lateral movement. a). Refers to the space allowance; only one type of system is scored; if different systems were found on-farm, each system must be scored independently. b). If animals had constant access to an outside yard and they could be outside at the same time the maximum score was assigned, if the access was restricted and/or not all animals could go out, the score of 1 was assigned. If there was no access to a yard the score assigned was 0. c). Referred to any part, partition and bars susceptible to harm the animals; the teguments of animals were also checked to detect any sign of deviation from the normality. d). If animals could move easily the maximum score (3) was assigned. If the movements of the animals were very restrained and/or if they had extreme difficulties to rise/lie down, the score assigned was -0.5. e). Total days spent at grass per year. The total locomotion score (column 1) equals to the sum of columns a). b). c). d). and e).

**Table 2:** Indicators in the AWI of the "Social interactions" category and the definitions used for rating and their maximum individual score.

**Score**	**a). Space allowance**			**b). Grouping**	**c). Rest areas**	**d). Calving method**	**e). Weaning method**	**f). Outdoor access**	**g). Grazing time (days per year)**
	
	**Slats**	**Loose housing**	**Tether systems**						
							
	**(m^2^/AWU)^1^**	**(m^2^/AWU)^1^**	**Movement of tether (m)**						
							
3		> 7.5							
2.5		> 6.5							> 270
2		> 5.5		Family herd					> 230
1.5		> 4.5		Herd without bull					> 180
1	> 3	> 4	> 0/6/0.4	Same age No regroup		Separate pen Visual Contact	Visual contact Gradual	Yes All of the time	> 120
0.5	2<X^2^ < 3		> 0.4/0.3		Yes			Yes Partially	> 50
0	< 2	< 4	< 0.4/0.3	Minimal Regroup/age mix	No	Separate pen No visual contact	Visual contact Abrupt	No	
0.5				Frequent Regroup/age mix		In pen with other animals	No visual contact		

^1^Animal Weight Unit (AWU); 1 AWU = 500 kg live weight. X^2 ^represents the observed value (X) between 2 and 3 m^2^/AWU. In tether systems, the first figure refers to back and forth movements, the second to lateral movement. a). Refers to the space allowance; only one type of system is scored; if different systems were found on-farm, each system must be scored independently b). Family herd consist of suckler cows with male and female calves, heifers and steers of the same family and integrated bulls. The total social interactions score (column 1) equals to the sum of columns a). b). c). d). e). f). and g).

**Table 3:** Indicators in the AWI of the "Flooring" category.

Score	a). Type of floor	b). Cleanliness of floor	c). Type of flooring	d). Yard cleanliness	e). Grassland
3					
2.5	Straw >60 mm		Straw >60 mm		
2	Straw 30-60 mm		Straw 30-60 mm		
1.5	Woodchip/peat		Woodchip/peat		
1	Mats	Clean	Mats	Clean	Good conditions
0.5	Softer slats	Medium	Softer slats	Medium	
0	Concrete slats	Soiled	Concrete slats	Soiled	Average conditions
-0.5	Concrete	Very soiled	Concrete	Very soiled	Poor conditions

The definitions used for rating, and their maximum individual score for the flooring category. a). and c). Softer slats refers to slats that were softer than concrete (for example wooden slats). b). and d). Clean: no slurry/mud could be found in the pen (100 to 80% for the straw or woodchip/slurry ratio); medium: not more than 3 spots of slurry/mud could be found in the pen for slatted floors (79 to 60% for the straw or woodchip/slurry ratio) soiled; more than 3 spots of slurry could be found in the pen (59 to 40% for the straw or woodchip/slurry ratio); very soiled: the pen was covered with slurry/mud (less than 40% for the straw or woodchip/slurry ratio). e) Score was assigned after checking the paddock size and frequency of new paddock with regard to the size of the herd, boundaries, conditions of alleys and gaps, number of topping per year and frequency of grass reseed and presence of shelters. The total "Flooring" category score (column 1) equals to the sum of columns a). b). c). d). and e).

**Table 4:** Indicators in the AWI of the "Environment" category, the definitions used for rating and their maximum individual score.

Score	a). Natural light	b). Artificial light	c). Side openings	d). Draughts	e). Condensation	f). Noise	g). Grazing time (days per year)
3							> 270
2.5							> 230
2	Open fronted						> 180
1.5	Very light	Very light					> 120
1	Light	Light		None		No noise	> 50
0.5	Medium	Medium	Yes	Sometimes	Good	Moderate	
0	Dark	Dark	No	Often	Ok	Noisy	
-0.5	Very dark	Very dark		Always	Bad	Intense	

a). Open fronted animal houses were considered as optimal conditions for light. The percentage of window area with light directed to the animals compared with the total floor surface was measured. Very dark: 0% (no natural light), very light 15%. b). If no artificial light was present, the score assigned was -0.5. In case of neons or compact fluorescent lights (CFL's), if less than one light per 5m^2 ^was present the score assigned was 0. If 1 to 1.5 lights per 5 m^2 ^were present, the score assigned was 0.5; 1 was assigned if between 1.6 to 2 lights per m^2 ^were present and 1.5 was assigned if more than 2 lights per 5 m^2 ^were present. In case of halogen lights, 0 was assigned of there was less than one lamp per 15m^2^, 0.5 if there was between 1 and 1.5 lamp per 15 m^2^, 1 if there was between 1.6 to 2 lamps per 15 m^2 ^and 1.5 if there was more than 2 lamps per m^2^. c). Represents the presence or absence of side openings. d). Draughts were considered when air flow was greater than 0.2 m/s. e). Air humidity was assessed subjectively with the forearms: if no humidity was felt the maximum score was assigned, if humidity could be clearly felt the minimum score was assigned. f) Noise of the fans and ventilation systems were assessed subjectively, the maximum score was assigned if no ventilation system was present. If a ventilation system was present and the noise started to be irritating for the ear, the minimum score was assigned. The total environment score (column 1) equals to the sum of columns a). b). c). d). e). f). and g).

**Table 5:** Indicators in the AWI of the "Stockpersonship" category, the definitions used for rating and their maximum individual score.

Score	a). Trough cleanliness	b). Outdoor water trough cleanliness	c). Feed cleanliness	d). Equipment	e). Cleanliness of animals	f). Lameness	g). Diseases	h). Background	I). Interest of the farmer
1	Clean	Clean	Clean	Good			None	Family	High interest
0.5	Medium	Medium	Medium	Medium	Clean	< 5%	Few mild		Average interest
0	Insufficient	Insufficient	Insufficient	Defects	Medium	5 to 10%	Few severe	Other	Low interest
-0.5	Soiled	Soiled	Soiled	Bad	Soiled	> 10%	Many severe		Not interested

a). b). and c). The troughs were considered clean when the water was clear, no algae could be seen in the water and no mud/slurry was present on them. Troughs were considered medium when the water was clear but small amount of algae could be found and/or few spots of mud/slurry of less than 2 cm of diameters were present on them. They were considered insufficient if the water started to be blurred but it was still possible to see through it, if the amount of algae was preponderant and if many spots of less than 2 cm in diameter of mud/slurry were present on them. They were considered soiled if the water was blurred and it was not possible to see through it, if algae colonized the troughs and if mud/slurry covered the troughs or many spots of more than 2 cm of diameter were found. e) Clean animals were covered with less than 10% of slurry/mud, medium between 11 and 20% and soiled over 20%. g) The list of diseases and symptoms consisted of: mild diseases (scours, worms, parasites) and severe diseases (bovine viral diarrhoea (BVD), bovine respiratory disease (BRD), Johne's disease, tuberculosis, leptospirosis and black leg). A maximum score of 1 was assigned if no disease was reported. A score of 0.5 was assigned if up to 2 mild diseases or symptoms were reported. A score of 0 was assigned if the presence of one or two severe diseases or more than two mild diseases were reported. A negative score of -0.5 was assigned if the presence of more than 2 severe diseases was reported or 4 mild diseases were reported. i) This indicator is subjective and the interest of the farmer was assessed using a questionnaire and face-to-face interview. The total Stockpersonship score (column 1) equals to the sum of columns; a). b). c). d). e). f). g). h). and i).

### AWI score

For each category, the indicators were evaluated and the farm was scored. The score for each indicator within a category was summated to give a category score. The category scores were then summated to give an AWI. The minimum score possible was -11.5 and the maximum score was 46, with a range of 57.5 points. The raw score was transformed into a relative score.

AWI = (Locomotion score + Social interactions score + Flooring score + Environment score + Stockpersonship score + 11.5) × 100/57.5. Farms were rated by means of ranks. The same ranking scale was used as that used with the TGI35L/2000. The animal welfare was considered as "inadequate" (IA) between 0 to 15% of the AWI maximum score, "adequate" (A) from 16 to 30%, "satisfactory" (S) from 31 to 50%, "good" (G) from 51 to 60%, "very good" (VG) from 61 to 75% and "excellent" (E) above 75% [5].

### Statistical analysis

The AWI and the category scores were tested for normality using a Shapiro-Wilk test (Genstat 11^th ^edition, VSD UK). The Student t-test for unpaired samples (Genstat 11^th ^edition) was performed to evaluate statistical differences in AWI between full-time and part-time farmers. The Student test for paired samples was used to investigate the differences between the AWI's of the first and second visits. Mann-Whitney tests were performed to determine the differences in individual indicator scores (not continuous variable) and Spearman's rank correlations were performed to identify the correlations of the number of animals and the interest of the farmers with the other indicators of the AWI. A Principal Component Analysis (PCA) was performed (DECISIA SPAD 6.5) with the different indicators of the AWI. When two indicators were highly correlated (Eigen value > 0.7 in the correlation matrix), the indicator with the least correlations was selected, then a second run of the PCA was conducted which included the illustrative data as described by Mazurek [14]. The PCA was performed using DECISIA SPAD 6.5 software using the COPRI procedure. Twenty-seven variables were entered as active continuous variables. Two illustrative continuous variables were also added (Table 6). The number of components was selected using the step third differences and Anderson's Laplacian intervals. A hierarchical classification was performed (using the Parti-Decla procedure of SPAD 6.5) in order to see if categories of farms could be determined and what was influencing them.

**Table 6:** Active and illustrative variables used to calculate the Principal Component analysis (PCA).

Active variables	Minimum score	Maximum score
Space allowance	0	2
Injurious protrusions	-0.5	0
Ease of locomotion	0	1
Grazing time	2.5	2.5
Age/group mixing	-0.5	1.5
Calving method	-0.5	1
Weaning method	-0.5	2
Type of floor	-0.5	2.5
Cleanliness of floor	0	1
Cleanliness of yard	0	1
Grassland	0.5	1
Natural light	0.5	2
Artificial light	-0.5	1.5
Side openings	0	1
Draughts	-0.5	1
Condensation	0.5	1
Noise	0.5	1
Water cleanliness	0	1
Water trough (cleanliness) outdoors	0.5	1
Feed cleanliness	0.5	1
Equipment	-0.5	1
Cleanliness of animals	-0.5	1
^1^Lameness	-0.5	1
Health	-0.5	1
Background	0	1
Interest of the Stockperson	0	1

**Illustrative variables**		

Number of animals	15	1000
Total score	17	36.5

The PCA show the minimum and maximum scores.

^1^Higher scores represent less lameness.

## Results

### Animal diets during housing

Animals had free access to grass silage. The mean *in vitro *dry matter (DM) digestibility was 603.8 g/kg DM (± 37.1 s.d.). The mean crude protein (CP) = 117.4 g/kg DM (± 10.8 s.d.). Silage was supplemented with concentrate feed having an *in vitro *ADF of 132.3 (± 17.3 s.d.) g/kg DM and CP was 155.4 g/kg DM (± 9.6 s.d).

### Farms status

**Figure 1:**
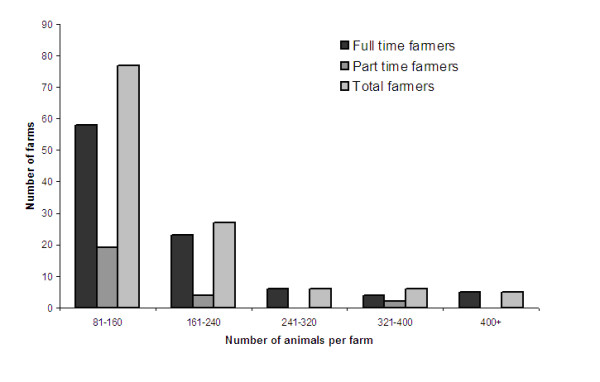
**Distribution of the total number of cattle on farms**. General mean = 131 animals per farm. First quartile corresponds to 59 animals, median to 100 animals and the third quartile to 150 animals. FT = full-time farmers; PT = part-time farmers; Total farmers (FT + PT).

Sixty-four percent (n = 125) of the interviewed farmers were full-time whereas 36% (n = 69) were part-time (Table 7). The total number of cattle per farm ranged from 15 to 1000 with a mean of 131 ± 9.9 (s.d.). Part-time farmers had a mean of 80 ± 7.7 (s.d.) animals and ranged between 17 and 370 animals per farm, while full-time farmers had 160 ± 13.5 (s.d.) animals per farm with a range between 15 and 1000 animals (Figure 1). Medians were respectively 61 and 120 animals. Part-time farmers owned a mean of 47 hectares while full-time farmers owned a mean of 76 hectares (P < 0.001).

**Table 7:** Number of full-time (FT) and part-time (PT) farmers and their respective category scores.

	FT	PT	P-values
Number of Farmers	125	69	
Mean number of animals	160 ± 13.5	80 ± 7.7	P < 0.001
**AWI score**	65 ± 7.0%	65 ± 7.0%	NS
Locomotion category score	54 ± 12.0%	55 ± 12.0%	NS
Social interactions category score	48 ± 12.0%	52 ± 11.0%	P = 0.001
Environment category score	88 ± 7.0%	87 ± 7.0%	NS
Flooring category score	50 ± 15.0%	48 ± 12.0%	NS
Stockpersonship category score	88 ± 9.0%	86 ± 9.0%	NS

The values are expressed as mean number of animals (± s.d) and mean scores (± s.d.; % of maximum score achievable in each category) of the AWI and each category.

NS = not statistically significant.

### AWI distribution

**Figure 2:**
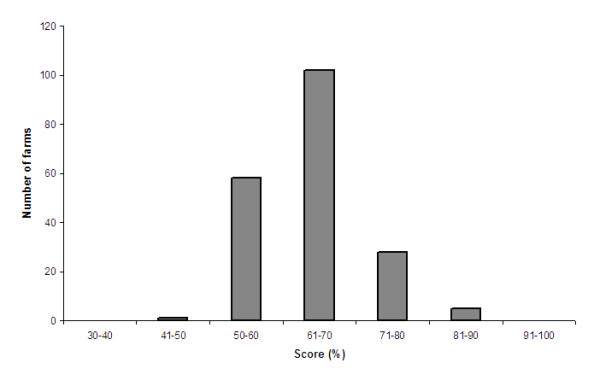
**Distribution of the AWI**. The AWI ranged from 54% and 83% with a mean of 65% (s.d. = 6%) of the maximum score.

**Figure 3:**
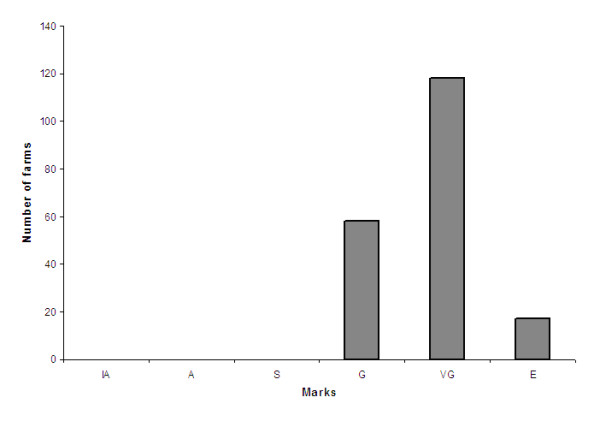
**Distribution of the welfare ranks (marks) of the farms**. IA = inadequate; A = adequate, S = satisfactory (1 farm); G = good (58 farms), VG = very good (118 farms); E = excellent (17 farms).

The score for each category was calculated (Table 7). The AWI ranged from 54% to 83% of the maximum score with a mean of 65% (s.d. = 7%) (Figure 2). The mean "locomotion" score was 54%. The mean "social interactions" score was 50%. The mean "flooring" score was 49%. The mean "light and air" score was 88%. The mean for the "Stockpersonship" score was 87%. The overall AWI ranged from "satisfactory" to "excellent" with a large majority (70%) of the farms rated as "Very Good" or "Excellent". The categorization of welfare status (inadequate, adequate, good, very good and excellent) is shown in Figure 3. No farm was scored as "inadequate" or "adequate". One farm was graded as "satisfactory", 58 farms were graded as "good", 118 farms were scored as "very good" and 17 farms were scored as "excellent".

The AWI scores were not different (P > 0.05) among the part-time and full-time farmers. The "social interactions" score was 48% for full-time farmers and 52% for part-time farmers and were considered as "satisfactory" for the full-time farmers and "good" for the part-time farmers. Part-time farmers had better "social interaction" category scores (P = 0.001). For individual indicators, full-time farmers had better scores for cleanliness (P = 0.03) of the animals and had less lame animals (P = 0.01) and had a tendency to have better "level of interest" scores (P = 0.052). Part-time farmers had better scores for the "grouping" (P < 0.001) and "weaning" (P = 0.03) indicators (Table 8).

**Table 8:** Significant differences in the individual indicators of the AWI between part-time and full-time farmers.

Category	Indicator	Significance	Ranks
Social	Grouping	P < 0.001	PT > FT
	Weaning method	P = 0.03	PT > FT

Stockpersonship	Cleanliness of animals	P = 0.03	FT > PT
	Lameness	P = 0.01	FT > PT
	Interest	P = 0.05	FT > PT

Part-time (PT) farmers; Full-time (FT) farmers.

### AWI/TGI35L/2000 comparison

A significant difference (P < 0.001) was found between the AWI and the TGI35L/2000. The TGI35L/2000 scores were lower (mean of 59 ± 7% (s.d.)) than the AWI scores (mean of 65 ± 6% (s.d.)). No difference (P > 0.05) was found for the locomotion category. A significant difference was found for the social category with mean scores of 37 **± **9% (s.d.) for the TGI35L/2000 and 53 ± 8% (s.d.) for the AWI, respectively. No significant difference (P > 0.05) was found between the two indices for the flooring category. A significant difference was found for the environment category with mean scores of 63 ± 11% (s.d.) for the TGI35L/2000 and 88 ± 7% for the AWI. A significant difference (P < 0.001) was found for the Stockpersonship category with 83 ± 6% (s.d.) for the TGI35L/2000 and 92 ± 9% (s.d.) for the AWI. Two farms went from "very good" rating with the TGI35L/2000 to "excellent" rating with the AWI. Five farms went from "good" rating with the TGI35L/2000 to "very good" rating with the AWI. Eight farms went from "satisfactory" rating with the TGI35L/2000 to "good" rating with the AWI.

### Statistical correlations

The number of animals was significantly correlated with the "health" score (R_s _= -0.8, P < 0.001), the "social interactions" category score (R_s _= -0.35, P < 0.001), the "grouping" score (R_s _= -0.32, P < 0.001), the "weaning method" score (R_s _= -0.23, P < 0.001) and the AWI score (R_s _= -0.21, P = 0.001). Lower correlations between the number of animals and other indicators were also found and presented in Table 9. The interest of the farmer was correlated with the "Stockpersonship" category score (R_s _= 0.67, P < 0.001), the "feeding space cleanliness" score (R_s _= 0.62, P = 0.012), the "floor cleanliness" score (R_s _= 0.47, P = 0.01), the "outdoor water cleanliness" score (R_s _= 0.44, P = 0.001), the "lameness" (a higher score indicates less lameness) score (R_s _= 0.0.43, P < 0.001), the AWI score (R_s _= 0.42, P < 0.001) and the "health" score (R_s _= 0.42, P = 0.023). Lower correlations between the interest of the farmer and other indicators were also found and presented in Table 10.

**Table 9:** Correlations between the total number of animals on-farm and the AWI, the category scores and the scores for the individual indicators.

Indicators	Significance	**R**_**s**_
AWI	P = 0.001	-0.21
Number of hectares	P = 0.001	0.17
Calving method	P = 0.04	-0.13
Type of floor	P = 0.04	-0.13
Locomotion score	P = 0.03	-0.14
Noise	P = 0.03	-0.14
Natural light	P = 0.02	-0.15
Space allowance per animal	P = 0.007	-0.19
Weaning method	P < 0.001	-0.23
Grouping	P < 0.001	-0.32
Social interactions score	P < 0.001	-0.35
Health	P < 0.001	-0.80

**Table 10:** Correlations between the interest of the Stockperson and the AWI, the category scores and the scores for the individual indicators.

Indicators	Significance	**R**_**s**_
AWI	P < 0.001	0.42
Stockpersonship score	P < 0.001	0.67
Feed cleanliness	P = 0.012	0.62
Cleanliness of floor	P = 0.01	0.47
Outdoors - water trough cleanliness	P = 0.001	0.44
Lameness (less)	P < 0.001	0.43
Health	P = 0.023	0.42
Cleanliness of animals	P = 0.003	0.40
Artificial light	P = 0.025	0.37
Weaning	P = 0.023	0.35
Space allowance per animal	P = 0.016	0.33
Environment Score	P = 0.009	0.30
Locomotion score	P = 0.023	0.29
Social score	P = 0.026	0.27

### AWI stability

No significant difference (P > 0.05) was found between the scores of the first visit and the second visit and a significant correlation was found between the scores (r = 0.86, P < 0.001).

### Principal component analysis (PCA) and hierarchical classification

**Figure 4:**
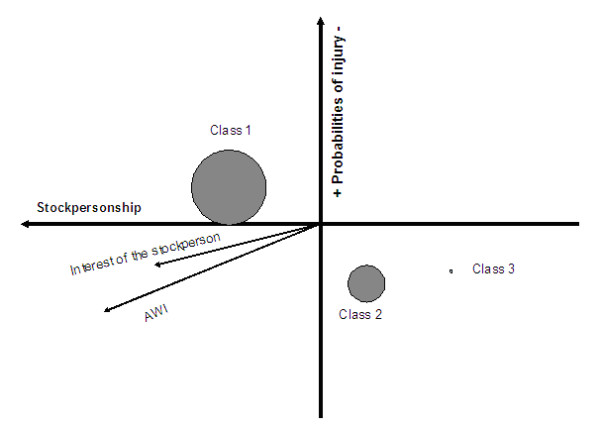
**Representation in the principal plan of the PCA of the 3 classes obtained with the hierarchical classification**. The first factor, "Stockpersonship" represented 11% of the variance. The second factor, "health and probabilities of injury", represented 9% of the variance. Class 1 (mean AWI = 66%, higher interest of the farmer) n = 130; Class 2 (mean AWI = 62%, lower interest of the farmer) n = 59; Class 3 (mean AWI = 54%, lowest interest of the farmer) n = 5.

Two components were retained to be described using Anderson' Lapacian intervals limits. The two first components represented 20% of the data variation. The first factor was described by Stockpersonship (indicators relative to cleanliness) and represented 11% of the variation. The second factor was described by the animals' health and probabilities of injuries (the indicators correlated to this factor were: "health", "ease of locomotion", and "injurious protrusions") and represented 9% of the variation. The first 10 factors (61% of the total variation) were used to calculate the classes for the hierarchical classification. Three classes were found within the hierarchical classification. The first class corresponded to farms with the best mean AWI (66%). The mean number of animals for this class was the same as the general mean. The first class regrouped clean farms with a good environment, and with a higher interest of the farmer than the average (0.82 against 0.71 for the general mean). This class represented 130 farms. The second class corresponded to farms that had a number of animals equal to the general mean. The AWI was the second in rank with 62% as an average. The class was characterized by better floor type and better natural light than the general mean but more injurious protrusions and worse ease of locomotion than the general means. They were also characterized with more diseases than the average. The interest of the farmer was lower than the general mean with 0.51 against 0.71 for the general mean. This class corresponded to 59 farms. The third class corresponded to five farms that had a lower AWI than the general mean (56%). It was correlated with lower Stockpersonship resulting in dirtier conditions and more diseases than the general mean. The mean number of animals for this class was similar to the general mean (Figure 4).

## Discussion

In agreement with the findings of Bartussek [4], it was possible to define an on-farm welfare score with the AWI. Although animal based indicators are more likely to be a better assessment of animal welfare than environment-based indicators [15], it is not always possible to evaluate them on farm because they are demanding in time and labour inputs from the farmer. Because of these limitations, the indicators that were measured in the present study were mainly environmentally based. Animal based and health indicators were also included. All indicators proved feasible to assess and the stability of the scores between the two visits showed that the repeatability was excellent.

A comparison of scores was made between the AWI and the TGI35L. The AWI used indicators that were in the original TGI35L/2000 [5], some of these indicators were modified and new indicators were used in the present study. Some indicators from the TGI35L/2000 were not used, for example, the levels of CO_2 _and NH_3 _in the animal housing. The maximum score assigned for access to pasture in the TGI35L/2000 and outside yards was 1.5 and 3, respectively. In the Austrian system cows graze at pasture for a short period (usually less than four months) [5], but would have daily exercise. In contrast, in seasonal grass based systems in Ireland, beef production systems typically comprise of a grazing season (usually seven to eight months) followed by an indoor winter period [16,17]. In these systems, typically, the majority of calves are spring-born and they are allowed to continually nurse the dam at pasture until the end of the grazing season in autumn when they are weaned and generally housed indoors for a period of 4 to 5 months. Under the conditions of the present study it was necessary to modify the TGI35L/2000 to assess the conditions at pasture. The TGI35L/2000 was designed for Austrian production systems that are managed differently to the present study.

The present study confirmed the importance of the farmer by his level of interest. A strong positive correlation was found between the interest of the farmer and the AWI as the Stockpersonship score was correlated with the interest of the farmer. More generally, a greater level of interest was linked with less lameness (reported by the farmer), better cleanliness (equipment, flooring and animals), less diseases (reported by the farmer), better environment score, better artificial lighting due to better buildings, a better locomotion score, better weaning methods and better social interaction scores. The interest of the farmer was not correlated with the number of animals and this is in agreement with Hemsworth [11]. The level of interest of the farmer was assessed by a means of a questionnaire. It has been reported that the attitude of the Stockperson was also important for the animals' welfare [18]. It was not possible to observe the farmer while working for reasons of timing, however, this is an indicator that should be included in future welfare assessments.

It was reported in the literature that the background of the farmer is important in the detection of welfare problems [18], therefore the indicator "background" was included in the AWI assessments. The importance of the interest of the farmer in the management of animals well-being is well documented [4], thus the "level of interest" indicator was included in the AWI.

Human-animal interactions (HAR) are a common feature of modern intensive farming systems and these interactions have been reported to have marked consequences on animal productivity and welfare [11]. Research has shown that the role and impact of the Stockperson on animal performance and welfare should not be underestimated [11,19,20]. The classes were well separated within the first two axes of the PCA and showed that the Stockpersonship (11% of the variance) was the most discriminating factor to assess animal welfare followed by the health (9% of the variance) of the animals. For the three classes, the interest of the farmer entered into the characterization of the classes (higher interest for the best scores (Class 1 (mean AWI = 66%, higher interest of the farmer) n = 130) and lower interest for the lowest scores (Class 2 (mean AWI = 62%, lower interest of the farmer) n = 59). The number of animals for each class did not differ from the general mean. In the third class (Class 3 (mean AWI = 54%, lowest interest of the farmer) n = 5), the level of interest of the farmer was significantly lower than the general mean. The mean AWI of class 3 was significantly lower than the general mean. This is in agreement with the literature [11,18].

While observing the major influence of the Stockperson on the AWI, it is of interest the farming activity of the latter (full-time or part-time) did not have an influence on the AWI. The results showed that part-time farmers had better "animal social interactions" category scores than full-time farmers, which may be due to better weaning and calving scores. In the present study, two thirds of the farms were managed by full-time farmers and one third by part-time farmers. By full-time farmers it was implied that they required more than 0.75 labour units to operate [12]. Regarding the number of animals per farm, the upper quartile was 150 animals and the median was 100 animals. It was not possible to know if the time allocated to management of the animals was similar.

The present AWI method was easily applicable on-farm and can combine more parameters than the TGI making it applicable in broader conditions. The comparison between the first and second visit showed that no difference could be found in the AWI. Only 5 pilot farms were necessary to train the assessors. The evaluation of the different indicators allowed the inspection to last 15 minutes at housing and less than 5 minutes at grass. Health and lameness levels were reported by the farmers, assessing these indicators directly by the assessor would demand substantially more time unless the operators have access to records. In the TGI35L/2000 it is stated that 40 minutes should be sufficient to evaluate all indicators.

## Conclusions

The welfare, health and management of farm animals are important factors that need to be considered in order to maintain optimal animal welfare and increase consumer acceptance of animal production in the near future. It was shown that the interest of the farmer and the number of animals on-farm were important factors that influenced the overall AWI. The AWI is an easy and quick method that could be used in countries with similar farm management as in Ireland but further research is needed to validate the assessment and the weight of some subjective parameters. Ideally this should take the form of a comparison/validation with an animal based method.

## Competing interests

The authors declare that they have no competing interests.

## Authors' contributions

BE, MM, DJP, IV and MAC designed the study. MM, DJP performed the farm evaluations. MM, BE analyzed the data and prepared the manuscript. All authors read and approved the final manuscript.
